# Frugal Droplet Microfluidics Using Consumer Opto-Electronics

**DOI:** 10.1371/journal.pone.0161490

**Published:** 2016-08-25

**Authors:** Caroline Frot, Nicolas Taccoen, Charles N. Baroud

**Affiliations:** LadHyX and Department of Mechanics, Ecole Polytechnique, CNRS, Palaiseau, France; Texas A&M University College Station, UNITED STATES

## Abstract

The maker movement has shown how off-the-shelf devices can be combined to perform operations that, until recently, required expensive specialized equipment. Applying this philosophy to microfluidic devices can play a fundamental role in disseminating these technologies outside specialist labs and into industrial use. Here we show how nanoliter droplets can be manipulated using a commercial DVD writer, interfaced with an Arduino electronic controller. We couple the optical setup with a droplet generation and manipulation device based on the “confinement gradients” approach. This device uses regions of different depths to generate and transport the droplets, which further simplifies the operation and reduces the need for precise flow control. The use of robust consumer electronics, combined with open source hardware, leads to a great reduction in the price of the device, as well as its footprint, without reducing its performance compared with the laboratory setup.

## Introduction

As microfluidic methods begin to make their way towards off-the-shelf platforms and real world applications, the technical developments that have taken place in research labs need to be simplified in order to make them easier to manufacture and more economically viable. A natural way to obtain this enhanced performance is to integrate the microfluidics with devices from the micro-electronics industry, which benefit from a long development history and economy of scale. Although these devices are often complex and rely on proprietary hardware and software, recent advances in open-source hardware, such as the Arduino platform (www.arduino.cc), make it easier to divert the commercial devices for purposes other than their original aim. This recycling of widespread and inexpensive technologies provides a basis for *frugal innovation* in microfluidics, by which complex operations can be performed with modest means. From a complementary point of view, adapting the existing consumer devices to new technologies can also benefit the micro-electronics industry, by providing new applications in which the existing tools can be used [1, 2].

Here we focus on the production and control of droplets in a microchannel. Indeed, a wide range of methods has been demonstrated for the manipulation of microfluidic droplets, starting with the control of the microfluidic geometry [3–6]. Intelligent operations on droplets, however, require active forcing through an external field, such as an electric field [7, 8], heat from a focused laser [9, 10], or acoustic forces [11]. While the electrical manipulation is now used in several labs, the other active manipulation methods remain at the proof of concept stage.

Nevertheless, optical manipulation of droplets presents several important advantages: for instance, manipulating the drop with a laser beam deports the experimental complexity off of the microfluidic chip, therefore keeping it very simple and inexpensive to micro-fabricate. Second, holographic beam shaping [12] or the ability to scan the laser spot anywhere in the field of view [13] provide ways to parallelize the manipulation of drops over several regions of the microfluidic device [14, 15]. This allows a very large number of operations to be performed with a single laser source, contrary e.g. to electrical methods that require a different electrode for each operation. Finally, the laser power required to manipulate drops in microchannels is relatively modest but still leads to forces near the micro-Newton range [16], several orders of magnitude larger than the forces generated by electrical forces [8]. However, most laser manipulation experiments (e.g. [9, 17, 18]) have been performed with research-grade lasers, on optical benches and using high performance microscopes. This makes the lasers expensive and complex to set up.

In this study we show how the laser from a commercial DVD writer is capable of performing similar operations to those described previously, but with a far smaller footprint and much more cheaply. The DVD device is coupled with a microchannel applying the “confinement gradient” approach to microfluidics [19], which simplifies the fluidic aspects and increases the robustness of the behavior. Below we begin by describing the experimental setup. This is followed by a characterization of drop formation in the device. We then show how the optical forcing from the DVD laser is capable of derailing drops on a single rail or to sort drops along multiple rails.

## Experimental setup

**Fig 1:**
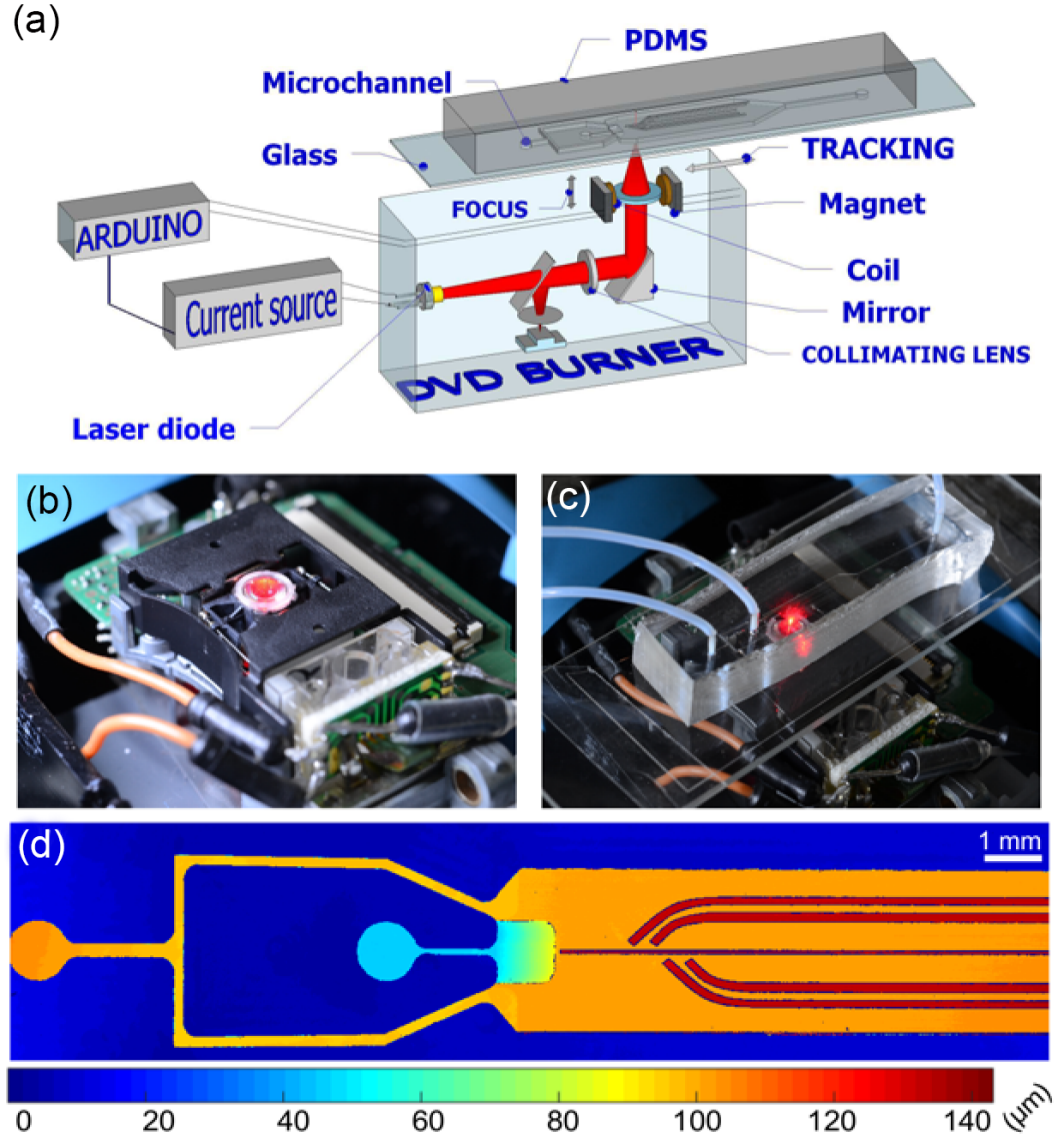
Coupling the microfluidics and the DVD writer. (a) Schematic of the microfluidic device on top of the DVD writer. The DVD focus and tracking positions are controlled through an Arduino card, which also controls the on-off switching of the laser. (b-c) Images of the DVD writer, without and with the microfluidic device in place. (d) 3D profilometry of the microfluidic circuit. The colors indicate the local channel depth, as shown on the color bar. The left-most entrance transports the continuous oil phase. The entrance on the right side is used to inject the water phase. The sloped region produces monodisperse drops passively. finally, a default central rail and four side rails are visible on the right side of the image.

The global setup and is sketched in Fig 1. It consists of a microfluidic channel coupled with a commercial DVD writer, which in turn is controlled *via* an Arduino interface and home-made electronics. In this section we describe each part of the experimental setup independently.

### Microfluidic device

The microfluidic device was made from a PDMS block, bonded to a thin (130 *μ*m) cover slip using plasma bonding. The PDMS was cast on an aluminium mold, which was fabricated using a micro-milling machine (Minitech). After sealing, the surfaces were made hydrophobic by incubating with Novec electronic coating (3M). Since all of the materials in the microfluidics were transparent to visible light, the DVD laser (wavelength 650 nm) was not absorbed and did not have the heating capability required for the thermocapillary control to be applied [16]. A simple way to remedy this was to draw a black mark on the bottom of the cover slip, below the region of interest, using a felt-tip marker. This black mark absorbed most of the laser power and was heated accordingly. The microfluidic device was then placed on a support glass slide directly above the DVD lens (Fig 1c), and the setup was observed through a fluorescence stereo-microscope while taking care to filter out the DVD laser wavelength (Thorlabs FES0550 shortpass filter, *λ*_cutoff_ = 550 nm).

The microfluidic geometry is based the confinement gradients, previously reported by our group: At the entrance region of the microfluidic device, the confinement gradient corresponds to a sloped ceiling, which is used to produce the droplets [19]. Further downstream, five “rails” are etched in the PDMS surface of the wide chamber. They correspond to regions of larger depth than the surrounding areas and they can guide the droplets in transverse directions with respect to the main oil flow direction [6]. For the rails, we reproduce the same geometric pattern initially used in Ref. [15]: In the absence of laser forcing, a default rail guides the drops down the centerline. When the laser is switched on near one of the side rails, the drops can derail and follow one of the oblique paths, each of which leading to a different position downstream. The side rails are sized to be slightly wider than the default rail so that a drop that feels both rails always follows the side rail. Conversely, the distance between the central and side rails is selected such that the unperturbed drops do not interact with the side rails. The device can therefore serve to sort drops of a well-defined and accurately controlled size.

### Controlling the laser

**Fig 2:**
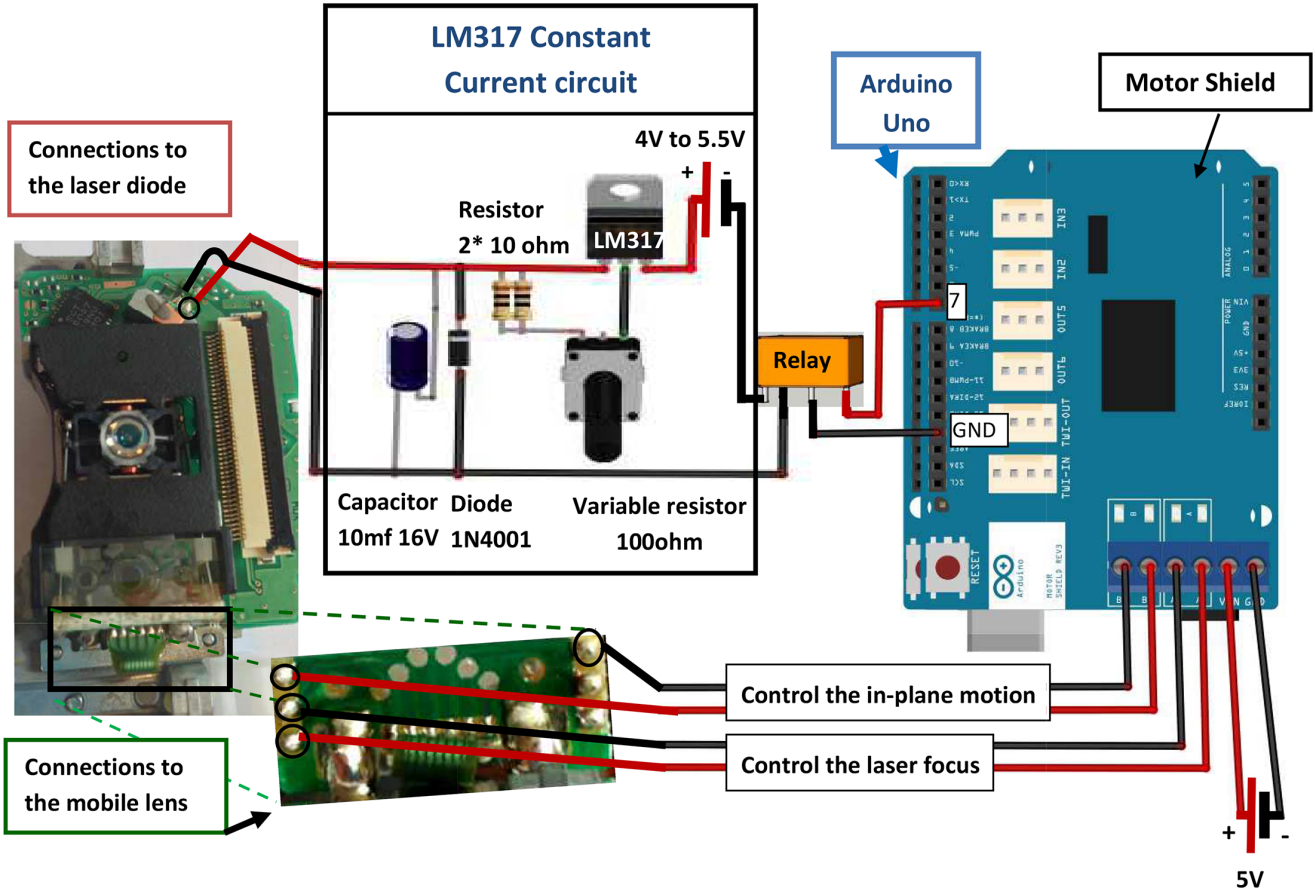
Controlling the DVD laser and lens. The DVD laser was controlled using a constant current source. The power output of the circuit was determined manually by turning the variable resistor. The mobile lens position was controlled through the Arduino motor shield. Its position could be programmed through software. Extreme care must be taken while manipulating the laser, as these are relatively high power lasers that can cause severe eye damage.

The electronic setup to control the laser position and power is shown in Fig 2. The experiments were performed using a commercial DVD writer from a desktop computer, controlled through open source hardware. The optical setup was used without modification: it consists of a laser beam that passes through integrated optical elements that include a fixed collimating lens on the laser diode, a dichroic mirror, and a mobile lens on top of the device (as sketched in Fig 1a). Note that it is very important to take necessary precautions while manipulating the laser, since it is very powerful and can cause eye damage. For this we built a cardboard housing surrounding the laser setup and covered the lens with a piece of paper in order to detect the laser, while keeping the power to a minimum.

It was not possible however to use the on-board electronics without deeper knowledge about how the DVD writer was made. For this reason we cut the connectors to the electronic circuits and soldered our own wires to them. However, each DVD device that we tested had different wiring diagrams, so it was necessary to determine where to connect the different inputs for each case. For this, it was useful to have a simple lab power supply (current limited in order to avoid burning the diode) and a digital signal generator.

The top lens of our DVD writer was mounted in a plastic housing and wrapped by two metal coils that allowed its motion to be controlled both in a horizontal plane and in a vertical plane. Determining which wires controlled the lens movement could be done by simple trial and error, for example by applying a sinusoidal signal from the signal generator, at an amplitude of about 1 V and oscillating at about 10 Hz. At these powers and frequencies, it was relatively easy to see the lens moving when the output of the signal generator was connected to the right wires.

Note however that the motion of the lens required some power and so it required a signal generator capable of producing it; the typical output from the Arduino uno card was not sufficient. We therefore used the Arduino UNO board coupled to the *motor shield* add-on, which acted as an amplifier. This provided one analog output to control the in-plane motion and a second one to control the laser focus (see Fig 2 for wiring details).

In turn, the laser diode was connected to a home-made current source circuit [20], which was powered by a lab power supply. One of the digital outputs of the Arduino board was used to switch the laser power on and off by controlling a relay, while the power was set manually by using the variable resistor. It was useful to connect the circuit to a voltage and current measurements, in order to verify its behavior in real time.

Determining how to connect the laser diode was done by visually inspecting the diode and seeing which wires lead to it. By using the power supply at 2.3 V, with the current limited to about 20 mA, we tested different pins until the laser began to shine. Once that was determined, it was easy to solder the wires coming from the Arduino to the connectors away from the diode itself, which also reduced the risk of burning the diode. Note that most DVD writers have the ability to read and/or write CDs. They therefore have two laser diodes that are usually set at right angles to each other, as well as the photodiode to collect the light. It is easy to tell the two lasers apart, since the CD laser shines in the infrared and is therefore not visible. In contrast, the DVD laser provides a bright red spot that can easily be detected.

The detailed final setup and the wiring diagram are shown in Fig 2. It is controlled through two Arduino codes, which are included as Supplementary Information: The first code provides a simple graphical interface to allow the user to click in different boxes on the screen in order to move and switch on the laser (S1 File). The second code uses the information obtained from the first code as input and sends the “move” and “switch on” commands to the lens and laser, respectively (S2 File). In this way, the laser power and its position could be imposed by a click of a mouse on a simple graphical interface.

### Calibrating the laser position

**Fig 3:**
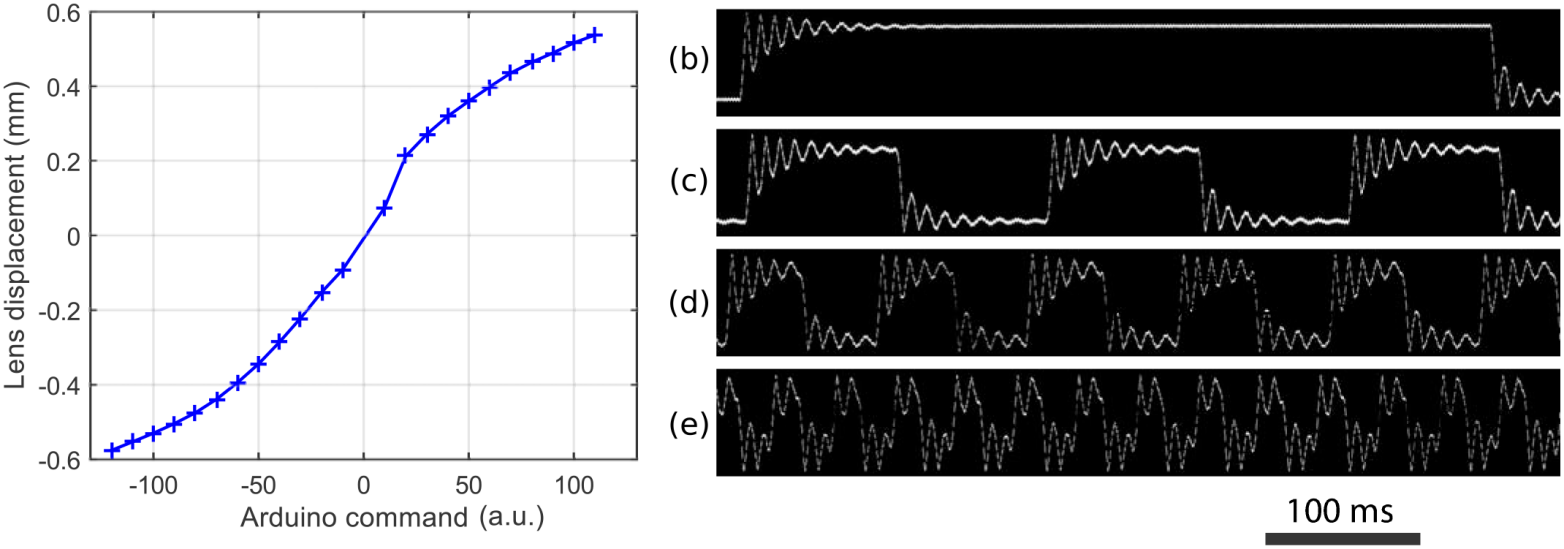
Calibration of laser forcing. (a) Position of the laser spot as a function of the command on the Arduino board, in the quasi-static regime. (b-e) Dynamic traces of the laser position as a function of time filmed through high speed camera. The laser was displaced over a distance of 1.05 mm with a duration of (b) 500 ms, (c) 100 ms, (d) 50 ms, and (e) 20 ms.

More precise determination of the motion can then be made by moving the lens, with the laser turned on, while covered with a piece of paper. This can be done under a microscope and observed with a digital camera, which allows precise measurements to be made from the image analysis (see Fig 3 below). Again, care must be taken to avoid shining the laser directly into the microscope, in order to avoid damaging the camera. It is also important to never look in the microscope eye-pieces when the laser is on.

Two parameters on the laser positioning were potentially limiting for our application: the maximum displacement of the lens and the frequency at which it could be moved. We tested these two parameters independently: the total displacement was found to be nearly 1.2 mm, which was well suited to the typical geometries of our microfluidic devices. A master calibration curve was generated (Fig 3a) to determine the electrical signal to set the in-plane and vertical displacement of the lens for each position of interest. Moreover, the focus of the laser spot was found to drift out of plane as the spot moved to extreme positions. This was visible by shining the laser on a flat sheet of paper and observing that the spot became wider as it moved away from the center. This artefact was corrected by applying a voltage on a different coil of the lens setup.

Regarding the system dynamics, temporal response was obtained by filming the laser spot on a sheet of paper using a fast camera (frame rate 4000 frames per second). The laser position thus obtained is shown in Fig 3b-3e, where a jump of 1.05 mm was performed with different delays between steps. We observed a ringing pattern that lasted around 100 ms for these large displacements, which is not limiting for our current experiments. In more demanding setups, this time can be reduced by applying passive or active (e.g. PID) control.

### Measuring the temperature inside the channel

Finally, the temperature within the microchannel was measured with a thermocouple (Radiospares type K, 75 *μ*m). This thermocouple was inserted into the wide region of the microchannel through the PDMS. The signal from the thermocouple was connected to a Max31855 amplifier, connected to the Arduino Uno board. For this experiment, the laser was at constant (high) power and the microchannel was mounted on an x-y-z translation stage. This allowed us to observe the temperature variations as the laser was focused or defocused, as well as measuring the temperature decrease away from the laser spot.

When the laser was focused properly on the dark spot, the observed maximum temperature was measured at 80°C. As the vertical position was varied, we observed a decrease to 55°C at a distance of 50 *μ*m vertically above best focus. In addition to these measurements, the horizontal variation in temperature was found to decrease to 50°C at a distance of 12 *μ*m away from the hottest spot. These measurements are similar to those obtained previously with the infrared laser which absorbed by a thin water layer directly [21]. They correspond to an equilibrium between the power source (in the form of laser heating) and heat dissipation through three-dimensional conduction in the liquid and solid masses.

## Droplet formation

Before addressing the droplet manipulation with the laser, we characterize the production and transport of the drops in the device. Although classic microfluidic methods have been shown to produce very monodisperse droplets, they tend to be well adapted for high-throughput production [22, 23]. At the low throughputs studied here (up to a few drops/second), droplets produced in a flow focusing device are too polydisperse. In contrast, using a confinement gradient to produce the droplets provides far more reproducible and robust droplet sizes [19]. This was found to be particularly true when the fluids were driven by a constant pressure source, rather than syringe pumps.

In the confinement gradient devices, the droplet generation and motion are determined by the 3D geometry, which is shown in Fig 1d. Published measurements indicate that the drop size is nearly independent of the flow rate of the dispersed phase when the outer fluid is stationary and the inner fluid is forced with a constant flux [19]. In that case however, the drops can only be transported until the end of the slope, which does not allow them to be extracted from the device further downstream. Here, both the dispersed and continuous phases are forced by a pressure controller (Fluigent): the oil through the left-most inlet and an aqueous-fluorescein solution in the second inlet.

**Fig 4:**
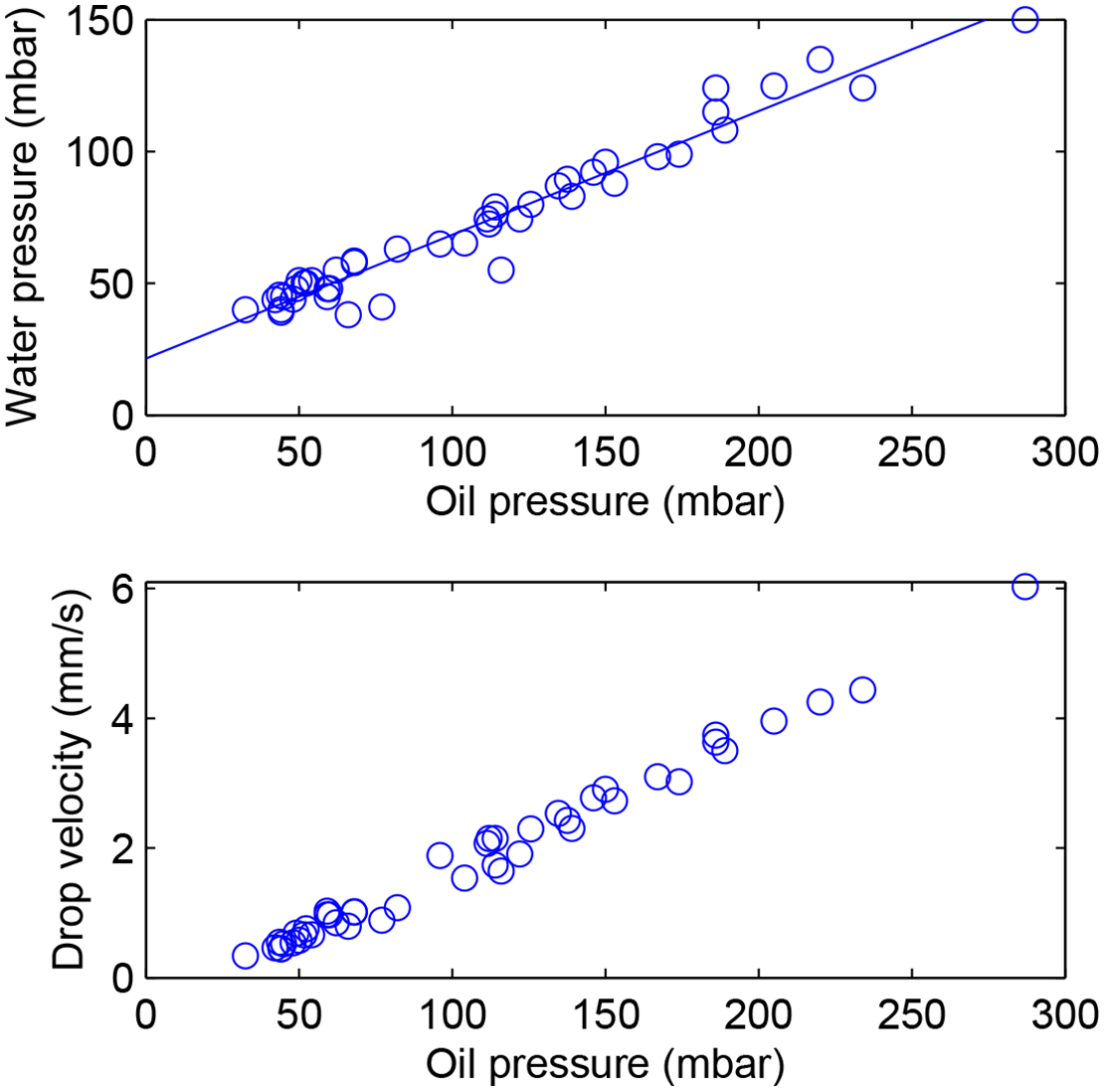
Drop size and velocity. (a) Value of the water inlet pressure that allows a constant droplet size, as a function of the imposed pressure on the oil inlet. (b) Velocity of the droplets, downstream of the sloped region, as a function of the imposed pressure on the oil inlet.

We therefore begin by measuring the water pressure that is required to produce droplets of the desired constant size, for a range of oil pressures. These measurements, shown in Fig 4a, display a linear dependence of the water pressure on oil pressure, with a slope of 0.47. This can be understood by calculating pressure distribution in the oil, by estimating the hydrodynamic resistances of the different channel segments [24]. This calculation shows that nearly half of the pressure loss takes place upstream of the junction, with the other half downstream. This explains the nearly 1/2 slope of the relation between the two pressures. More interestingly, the y-intercept gives *P*_*water*_ = 20 mbar, which is the required pressure to produce droplets even at zero oil flow rate. This value is in good agreement with the capillary pressure jump, due to the Laplace pressure at the curved interface as it exits the microchannel into the sloped region. Indeed, this Laplace pressure can be estimated as Δ*P*_*Laplace*_ ≃ *γ*(2/*h* + 2/*w*), where *γ* ≃ 30 mN/m is the oil-water interfacial tension and *h* = 40 *μ*m and *w* = 80 *μ*m are the height and width of the inlet channel, respectively. This yields Δ*P*_*Laplace*_ = 22 mbar, in close agreement with the measured value. Therefore for a given driving pressure on the oil inlet, the water pressure required to produce constant size droplets is given by the value of the pressure in the oil at the channel inlet plus the Laplace pressure jump.

Additionnally, the droplet velocity, as it interacted with the laser, was previously found to determine whether or not drops could be manipulated with the laser heating [16]. We therefore used image analysis to determine the velocity at which each droplet was travelling, in the vicinity of the first rail, for different oil pressures. The results are shown in Fig 4b. They show that the velocity of droplet movement also increases linearly with the oil pressure. As such, we can consider that the oil pressure is the relevant parameter to determine the operating conditions of the experiment.

## Derailing droplets

**Fig 5:**
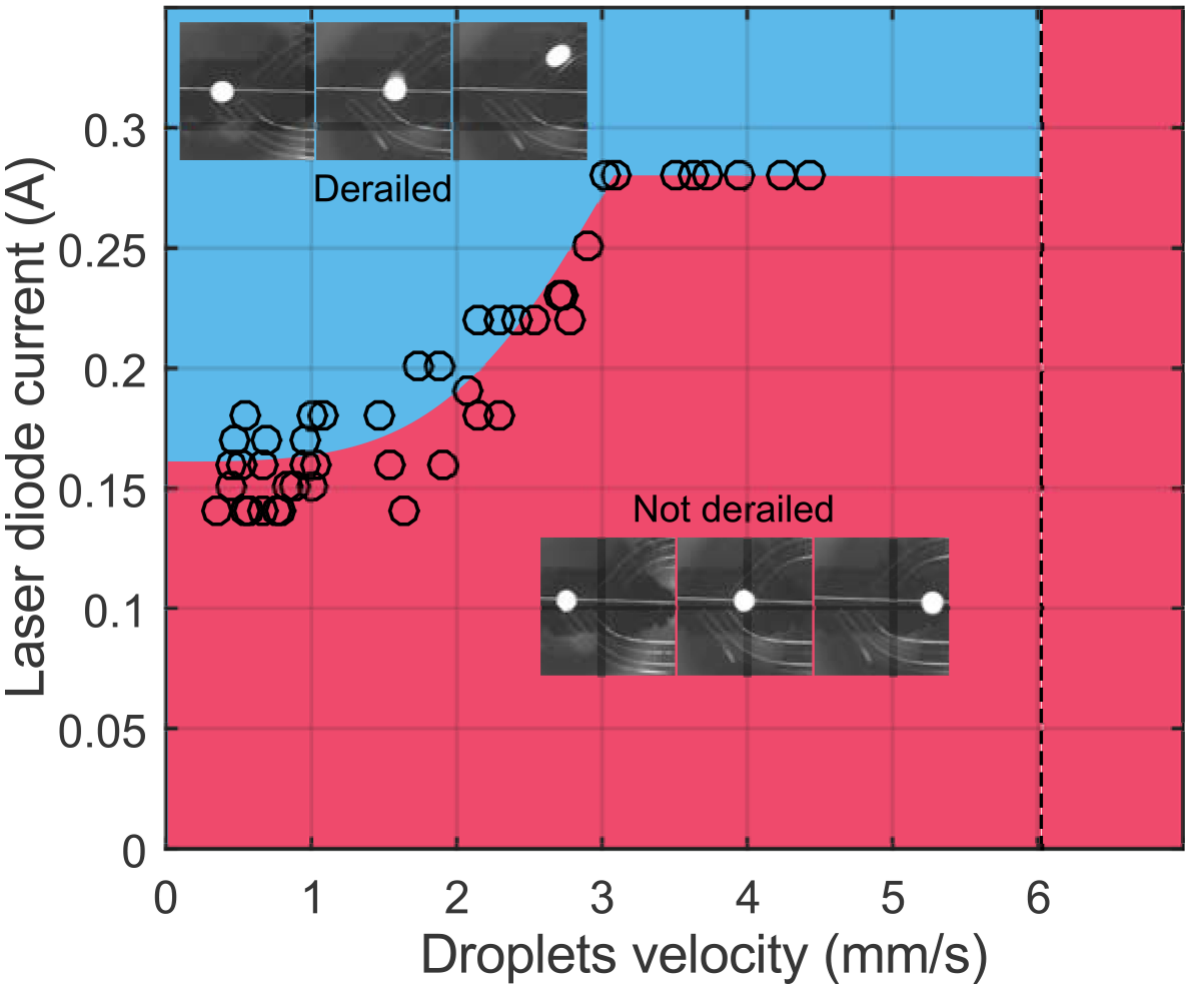
Required power for derailing drops. Minimum electrical current required to derail droplets onto the first rail. Faster moving droplets require a more intense laser to switch rails. The largest current achieved on this laser was 0.28 A. Drops beyond a velocity of 6 mm/s could not be derailed at this maximum value.

With the mechatronic and fluidic aspects calibrated, the ultimate test of the approach was to verify if the laser power was sufficient to derail a droplet from the default rail to one of the side rails. For this we selected a position for the laser focus near the first rail and recorded the fate of the droplets as they reached the hot spot. The droplet velocity was varied by controlling the pressure driving the oil; for each imposed pressure, we recorded the minimum current into the laser diode necessary to derail ten successive drops. The results are shown in Fig 5. Faster drops were found to require higher power to derail, until the maximum available current (280 mA) was reached for droplets going faster than 3 mm/s. Beyond a velocity of 6 mm/s, it was no longer possible to derail the drops with the current setup.

This electrical measurement was used as a surrogate variable to the optical power, to which we did not have access. However if we assume that the diode has a 25% efficiency and that the operating voltage is 2.8 V (measured), we estimate that the optical power is around 200 mW. These values are consistent with previous experiments [16], where the required laser power to stop a droplet in a microchannel was found to increase with the drop velocity. The infrared laser used by Verneuil et al. however was absorbed directly by the aqueous phase, so that the heating began only when the water-oil interface intersected the laser spot. This is not the case here, since the DVD laser heats the black mark on the glass slide. So the droplets begin to feel the presence of the hot region, by thermal conduction, before reaching the laser focus positions. In both cases, nevertheless, fast-moving droplets cannot be deviated and instead remain on the central rail despite the presence of the hot region.

## Sorting drops along multiple rails

**Fig 6:**
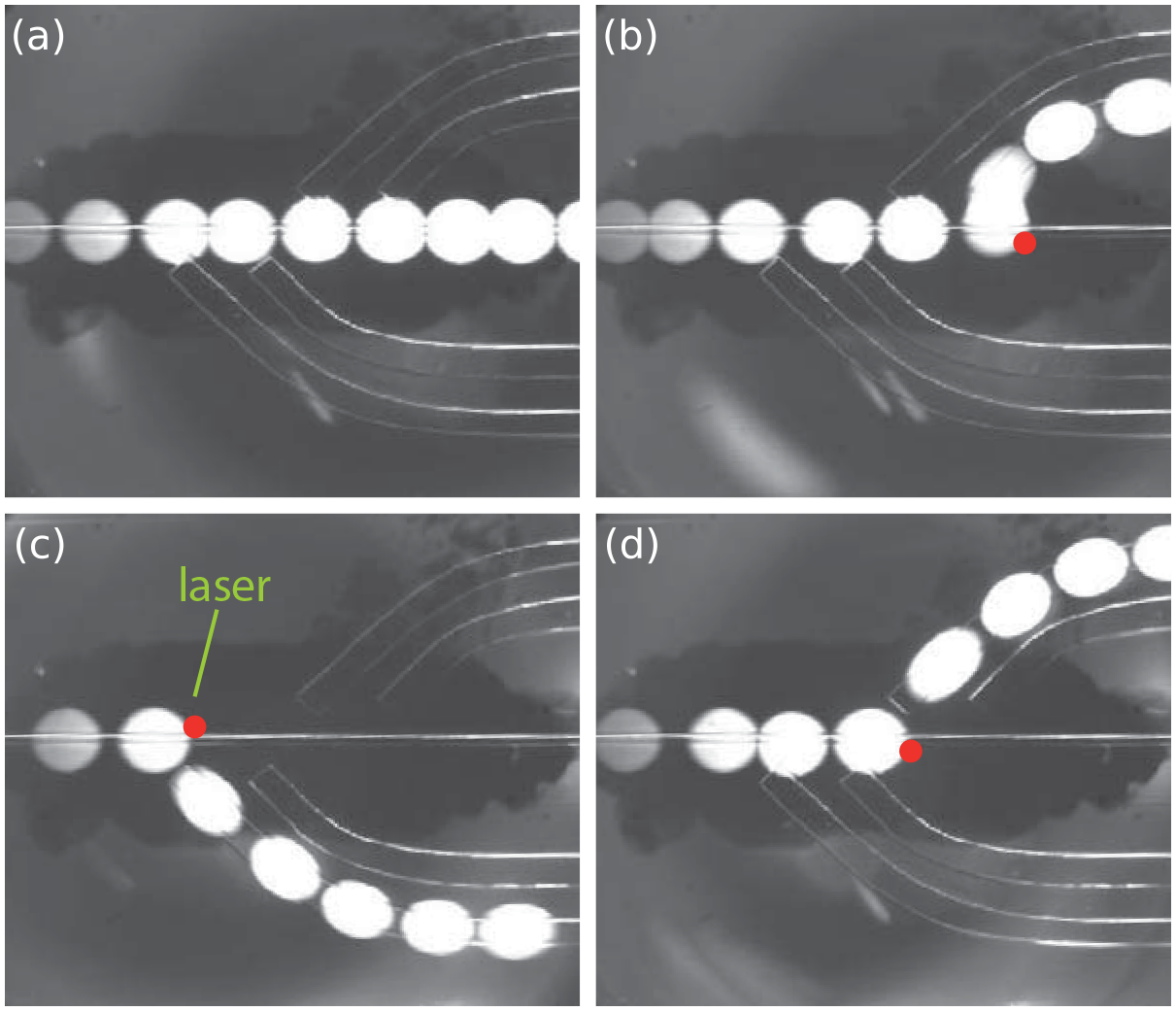
Multiple sorting positions. Sorting droplets onto different rails by controlling the position of the laser lens. Each panel shows a superposition of images for a single drop in the device. (a) If the laser is off, the drop remains on the default central rail. (b-d) Different laser positions force the drop to jump on different rails, which lead to different regions downstream.

The strength of this frugal microfluidic device is best demonstrated by showing how it can be used to sort drops along four different rails, by controlling both the position and power of the DVD laser. A demonstration is shown in Fig 6, and the accompanying video (S1 Video). In the absence of a laser, the droplets follow the central default rail and they remain on it until they leave the device. Then by moving the laser spot to different locations, different rails can be selected and the droplets can be transported to different parts of the wide channel.

Here, the oblique orientation of the rails serves to amplify the lateral distance between drops after they are derailed. As such, a small perturbation of a few microns in the droplet positions, due to the laser heating, translates into a distance of several mm at the downstream end of the channel. Drops that are thus separated can be extracted from different exits or subjected to different treatments.

## Discussion and outlook

It is common for technologies to advance by adding features and complexity, while increasing the cost over previous versions. The idea in frugal innovation is to reverse this trend by simplifying the technologies and reducing their cost, while preserving the important performance characteristics. This often requires increasing the inherent robustness of the products in order to reduce their reliance on external control features or frequent maintenance.

In the case of droplet microfluidics the complexity emerges from the nonlinear behavior of drops in microchannels and from the necessary lab equipment to detect their contents and perform manipulations on them. Here we simplify the requirements along both axes: (i) By implementing droplet microfluidics based on confinement gradients we ensure that the droplet behavior is determined by the channel’s 3D geometry, which makes it robust to perturbations in the forcing. (ii) By performing the manipulations with a commercial DVD writer we reduce the complexity of the experimental setup, its cost, and its footprint.

This frugal approach can be pushed further by using the signal from the photodiode, in order to detect the presence of the droplet, or even by using a blue-ray reader to excite the fluorescence within the drops. Such operations are simple to implement in principle and would remove the need for a microscope for some applications. Moreover, improved control schemes of the laser power and position would also greatly enhance the temporal response of the system and thus the potential throughput of the device.

Finally, note that the device demonstrated here is indeed very cost-effective: the DVD writer has a cost around 20 Euros, to which we added 50 Euros for the Arduino and other electronics. The microfluidic chip is fabricated by casting PDMS on a mold fabricated with a milling machine. Such a chip, which is completely passive, can be fabricated in a wide range of polymers or glass, which can further keep the costs down. Moreover, since the dynamics within the device are dominated by the 3D geometry, lower quality pressure control can be used without reducing the device operation, which removes the need for a programmable pressure source. Finally, the lab microscope can be replaced by an inexpensive USB microscope for many applications. As such, the total experiments that we demonstrate here can be performed for a few hundred euros, which is well suited for resource-limited settings or for teaching labs. By the same token, they demonstrate that an optical based manipulation of droplets for Lab on a chip applications is within easy reach for industrialisation.

## Supporting Information

S1 File *Processing* code for the mouse input. Listing of the *Processing* code that acquires the mouse clicks and translates them into preset positions of the laser. (https://processing.org/.)
(PDF)

S2 File Arduino code for moving and power-cycling the laser. Listing of the Arduino code that controls the laser position and power. At the mouse click, the code selects the position of the lens and switches the laser on for 100 ms, before switching it back off. (https://www.arduino.cc/.)
(PDF)

S1 Video Movie of the device operation. A train of droplets arrives at the sorting region, as the laser is displaced and power-cycled manually to sort them. The drops are guided on the different side-rails depending on the laser forcing.
(AVI)
