# Supplementary material for: Frugal Droplet Microfluidics Using Consumer Opto-Electronics
Source: PLoS One. 2016 Aug 25;11(8):e0161490. doi: 10.1371/journal.pone.0161490 (PMC4999286; doi:10.1371/journal.pone.0161490)

PROCESSING

```
import processing.serial.*;//communication
Serial myPort;  // Create object from Serial class

void setup(){
 size(800,300);// screen size
 String portName = Serial.list()[1]; //change the 0 to a 1 or 2 etc. to match your port
  myPort = new Serial(this, portName, 9600);}

void draw(){  // draws rectangles
 fill(200,0,100); rect( 100 , 100 ,100,100 );
fill(200,0,500); rect( 250 , 100 ,100,100 );
fill(500,0,10); rect( 400 , 100 ,100,100 );
fill(800,0,500); rect( 550 , 100 ,100,100 );

if (mousePressed && (mouseX > 100 && mouseX < 200 && mouseY > 100 && mouseY < 200 )) {
    myPort.write('0');
   println("2");
    }
        if (mousePressed && (mouseX > 250 && mouseX < 350 && mouseY > 100 && mouseY < 200 )) {
         myPort.write('1');
          println("2");
       }
         if (mousePressed && (mouseX > 400 && mouseX < 500 && mouseY > 100 && mouseY < 200 )) {
        myPort.write('2');
        println("2");
      }
         if (mousePressed && (mouseX > 550 && mouseX < 650 && mouseY > 100 && mouseY < 200 )) {
          myPort.write('3');
println("2"); }}
```

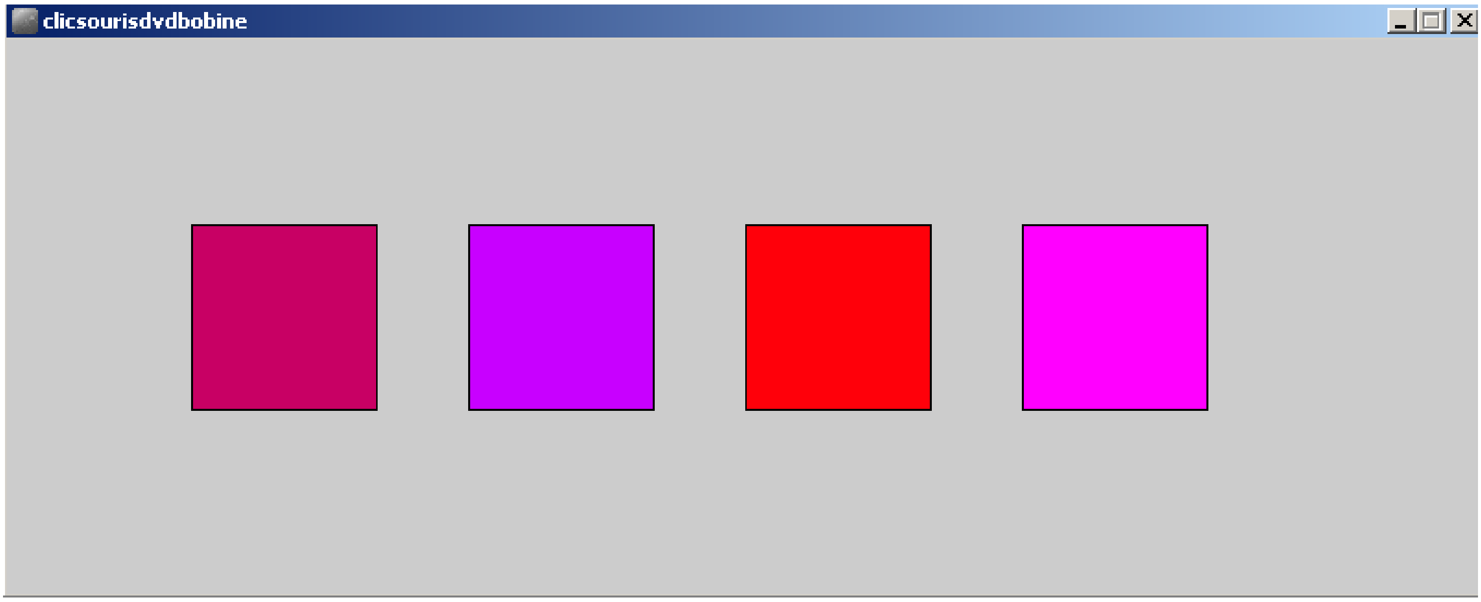

Supplement: S1 File — Listing of the Processing code that acquires the mouse clicks and translates them into preset positions of the laser. (https://processing.org/.) (PDF) [file pone.0161490.s001.pdf]
