# Supplementary material for: Frugal Droplet Microfluidics Using Consumer Opto-Electronics
Source: PLoS One. 2016 Aug 25;11(8):e0161490. doi: 10.1371/journal.pone.0161490 (PMC4999286; doi:10.1371/journal.pone.0161490)
Supplement: S2 File — Listing of the Arduino code that controls the laser position and power. At the mouse click, the code selects the position of the lens and switches the laser on for 100 ms, before switching it back off. (https://www.arduino.cc/.) (PDF) [file pone.0161490.s002.pdf]

```
char val; // Data received from the serial port
int val1;
void setup() {

//Relay
   pinMode(7,OUTPUT);
   pinMode(2,INPUT);
   pinMode(0,INPUT);

//moving the lens in Z
  pinMode(12, OUTPUT); //Z direction
  pinMode(9, OUTPUT); //Brake Z
  pinMode(3, OUTPUT); //Z Lens displacement (height)

//moving the lens in X
  pinMode(13, OUTPUT); //X direction
  pinMode(8, OUTPUT); //Brake X
  pinMode(11, OUTPUT); //X Lens displacement

  Serial.begin(9600); // Start serial communication at 9600 bps
 }

void loop() {
  digitalWrite(12, LOW); //bobine Z direction
  digitalWrite(9, LOW);   ////Disengage the Brake for Z

 //Communication with processing
  if (Serial.available())  { // If data is available to read,
     val = Serial.read(); // read it and store it in val

 if (val == '0')
     {        digitalWrite(7, HIGH);//relay closed
              digitalWrite(13, HIGH); // X Lens direction
              digitalWrite(8, LOW); ////Disengage the Brake for Z
              analogWrite(11,120); // X Lens displacement
              analogWrite(3,115);//Z Lens displacement (height)
              delay(100);
              digitalWrite(7, LOW);//relay opened
     }
     if (val == '1')
```

```
{       digitalWrite(7, HIGH);//relay closed
        digitalWrite(13,HIGH); // X Lens direction
        digitalWrite(8, LOW);   ////Disengage the Brake for Z
        analogWrite(11,40); // X Lens displacement
        analogWrite(3,90);//Z Lens displacement (height)
        delay(100);
        digitalWrite(7, LOW);//relay opened
}
if (val == '2')
{       digitalWrite(7, HIGH);//relay closed
        digitalWrite(13, LOW); //X Lens direction
        digitalWrite(8, LOW);   //Disengage the Brake for Z
        analogWrite(11,20);  //X Lens displacement
        analogWrite(3,90);//Z Lens displacement (height)
        delay(100);
        digitalWrite(7, LOW);//relay opened
}
if (val == '3')
{       digitalWrite(7, HIGH);//relay closed
        digitalWrite(13, LOW); //X Lens direction
        digitalWrite(8, LOW);  //Disengage the Brake for Z
        analogWrite(11,120);//X Lens displacement
        analogWrite(3,200);//Z Lens displacement (height)
        delay(100);
        digitalWrite(7, LOW);//relay opened
} }}
```
